# Antioxidant Supplementation in the Treatment of Aging-Associated Diseases

**DOI:** 10.3389/fphar.2016.00024

**Published:** 2016-02-12

**Authors:** Valeria Conti, Viviana Izzo, Graziamaria Corbi, Giusy Russomanno, Valentina Manzo, Federica De Lise, Alberto Di Donato, Amelia Filippelli

**Affiliations:** ^1^Department of Medicine and Surgery, University of SalernoBaronissi, Italy; ^2^Department of Medicine and Health Sciences, University of MoliseCampobasso, Italy; ^3^Department of Biology, University of Naples Federico IINaples, Italy

**Keywords:** vitamins, resveratrol, sirtuins, hormesis, oxidative stress

## Abstract

Oxidative stress is generally considered as the consequence of an imbalance between pro- and antioxidants species, which often results into indiscriminate and global damage at the organismal level. Elderly people are more susceptible to oxidative stress and this depends, almost in part, from a decreased performance of their endogenous antioxidant system. As many studies reported an inverse correlation between systemic levels of antioxidants and several diseases, primarily cardiovascular diseases, but also diabetes and neurological disorders, antioxidant supplementation has been foreseen as an effective preventive and therapeutic intervention for aging-associated pathologies. However, the expectations of this therapeutic approach have often been partially disappointed by clinical trials. The interplay of both endogenous and exogenous antioxidants with the systemic redox system is very complex and represents an issue that is still under debate. In this review a selection of recent clinical studies concerning antioxidants supplementation and the evaluation of their influence in aging-related diseases is analyzed. The controversial outcomes of antioxidants supplementation therapies, which might partially depend from an underestimation of the patient specific metabolic demand and genetic background, are presented.

## Introduction

Reactive oxygen species (ROS) comprise both free radicals such as superoxide (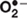), and non-radical species such as hydrogen peroxide (H_2_O_2_), (Weseler and Bast, 2010; Gülçin, 2012). These molecules, continuously produced in the cell, are involved in physiological events such as cell differentiation, primary immune defense, and signaling (Poli et al., 2004; Shah and Sauer, 2006; Gülçin, 2012). Indeed, some ROS such as H_2_O_2_ are versatile players of the molecular signaling machinery because they are small, highly diffusible, and can be rapidly generated and degraded (Gough and Cotter, 2011).

Both radical and non-radical ROS are pro-oxidant species capable of oxidizing in the cell different biomolecules (Sies, 1991), which leads to a sequence of chain reactions that may end up in molecular and cellular damage (Gülçin, 2012).

The balance between beneficial and detrimental effects of ROS is preserved in the cell by the activity of a complex array of non-enzymatic and enzymatic detoxification mechanisms collectively known as antioxidants (Sies, 1993; Bast and Haenen, 2015). Antioxidants are able to counteract, at relatively low concentrations, the damage induced in cells by ROS, thus protecting physiological targets such as lipids, DNA and proteins (Loguercio et al., 2012; Bast and Haenen, 2015). Noteworthy, antioxidants may also act indirectly by regulating redox-sensitive signal transduction pathways including transcription factors, and inhibition of poly (ADP-ribose) polymerase (PARP-1) (Weseler and Bast, 2010).

An imbalance of either the pro-oxidant and/or the antioxidant parties is at the origin of a complex physiological status known as “oxidative stress” (Davies, 1995; Conti et al., 2015a,b), which may be also favored by external sources, and by the presence of dietary compounds of pro-oxidant nature such as quinones (Gülçin, 2012).

Elderly people are more susceptible to oxidative stress due to a reduction in the efficiency of their endogenous antioxidant systems. Organs such as heart and brain, with limited replication rate and high levels of oxygen consumption, are particularly vulnerable to this phenomenon, thus explaining almost in part the high prevalence of neurological and cardiovascular diseases (CVD) in elderly (Ames et al., 1993; Stadtman and Berlett, 1997; Corbi et al., 2008).

A substantial body of literature reported an inverse correlation between serum or plasma total antioxidant capacity and both the onset and progression of several diseases, primarily CVD (Ciancarelli et al., 2012), but also diabetes (Opara et al., 1999), respiratory (Gumral et al., 2009) and neurological disorders (Schrag et al., 2013).

Consequently, antioxidants supplementation was suggested as a promising therapy in line with the general acceptance of the Free Radical Theory of Aging (FRTA) (Harman, 1956, 2006). First presented in Harman (1956), this theory is based on the assumption that lowering the global level of ROS in the body might retard aging, increase life span and be effective in preventing and treating aging-associated diseases (Sadowska-Bartosz and Bartosz, 2014). Further refinements of this theory addressed the roles of other activated oxygen species in aging in the more generalized Oxidative Stress Theory of Aging (OSTA) (Bokov et al., 2004; Muller et al., 2007).

This awareness resulted from one side in boosting in the scientific community the quest for novel natural or synthetic antioxidants (Donadio et al., 2015), and on the other in establishing several treatment strategies whose aim was to counterbalance oxidative stress by supplementing exogenous antioxidants, either singularly or in combination (Bouayed and Bohn, 2010).

However, the clinical expectations of antioxidants-based therapies have been frequently disappointed. The interplay between endogenous and exogenous antioxidants with the overall redox system in humans is very complex and represents a topical issue that is still under debate in the scientific community.

In this review a selection of recent clinical studies concerning antioxidants supplementation and the evaluation of their influence in aging-related diseases is analyzed (**Table 1**).

**Table 1:** Clinical studies performed with the main antioxidants.

Antioxidant	Disease	Primary results	Reference
Vitamin C	Type 2 DM and CAD	↑ Forearm vasodilator response	Antoniades et al., 2004
	DM	↓ Arterial blood pressure and improvement of arterial stiffness	Mullan et al., 2004
	IHD	Prevention of nitrate tolerance	Watanabe et al., 1998
Vitamin E	CAD	↓ Rate of non-fatal MI	Stephens et al., 1996
	CVD and DM	No effect on cardiovascular outcomes	Yusuf et al., 2000
	Vascular disease and DM	↑ Risk for HF	Lonn et al., 2005
	Prior MI	↑ Chronic HF in patients with LVD	Marchioli et al., 2006
	CAD	↓ Plasma biomarkers of oxidative stress and inflammation	Devaraj et al., 2007
Resveratrol	Hypertension and dyslipidemia	↓ Endothelial dysfunction	Carrizzo et al., 2013
	DM	Improvement of glucose control and insulin sensitivity	Liu et al., 2014
Coenzyme Q10	CAD	↑ Antioxidant enzymes activities and ↓inflammation	Lee et al., 2013
Vitamin E and C	Hypercholesterolemia	↓ atherosclerotic progression	Salonen et al., 2003
Vitamins C, E and β-carotene (alone or in combination)	CVD	No overall effects on cardiovascular events	Cook et al., 2007


*DM, diabetes mellitus; CAD, coronary artery disease; IHD, ischemic heart disease; CVD, cardiovascular disease; MI, myocardial infarction; HF, heart failure; LVD, left ventricular dysfunction.*

## Natural Antioxidants Used in Recent Clinical Studies

Many natural compounds have been considered, either singularly or in combination, for supplementation therapies. Among them, we devoted particular attention to a specific subset of molecules such as vitamin C, vitamin E, resveratrol, curcumin, hydroxytyrosol and coenzyme Q10.

Ascorbic acid is the main form of vitamin C in the human body and acts as the co-substrate for several enzymes that are important for the organism’s functioning. Its antioxidant activity relies on the ability to be reversibly oxidized to ascorbyl radical and then to dehydroascorbate (DHA) (Wells and Xu, 1994).

The distribution and the concentration of vitamin C in the organs depend on their specific ascorbate requirements and on the tissue distribution of sodium-dependent vitamin C transporter 1 and 2 (SVCT1 and SVCT2) (Figueroa-Mendez and Rivas-Arancibia, 2015).

Vitamin C (ascorbic acid) has different important roles in the cell; as a reducing agent and an antioxidant, ascorbate is able to react and inactivate ROS and, most importantly, reduces in membranes LDL and α-tocopheroxyl radicals to regenerate α-tocopherol (Vitamin E) (Chambial et al., 2013).

One of the several biological functions mediated by ascorbate is the enhancement of nitric oxide bioavailability, which is essential to preserve endothelial homeostasis (Carr and Frei, 1999). A recent metanalysis (Ashor et al., 2014) revealed that vitamin C supplementation counteracts endothelial dysfunction (ED), which is doubtless one of the major contributors for both the development and progression of CVDs (Versari et al., 2009; Conti et al., 2012b, 2013; Corbi et al., 2012; Zhang et al., 2014), suggesting a clinical impact of supplementation only in subjects at higher CVDs risk. Antoniades et al. (2004) found that a vitamin C supplementation of 2 g/day for 4 weeks increased forearm vasodilator response to reactive hyperemia in patients with combined diabetes (DM) and coronary artery diseases (CAD).

An interesting study by Mullan et al. (2002) showed that an oral administration of ascorbic acid (500 mg/day) for 1 month lowered blood pressure and reduced systemic arterial stiffness; conversely, other two randomized controlled trials failed to prove a blood pressure-lowering effect of vitamin C supplementation (Lovat et al., 1993; Ghosh et al., 1994).

However, the role played by vitamin C in aging-associated diseases has not been adequately investigated in clinical trials mainly because this antioxidant was often used in combination with other molecules (Watanabe et al., 1998; Salonen et al., 2003).

When referring to vitamin E, a family of 8 isoforms classified in two categories is considered: four saturated analogs (α, β, γ, and δ) called tocopherols and four unsaturated analogs indicated as tocotrienols, which differ for the methylation pattern (Cardenas and Ghosh, 2013). These molecules are hydrophobic fat-soluble compounds found in a variety of food sources such as corn oil, peanuts, vegetable oils, fruits and vegetables (Cardenas and Ghosh, 2013). Most of the studies presented in literature have been performed using α-tocopherol (Wallert et al., 2014; Hanson et al., 2015) that is considered the most active isomer within this group and the main hydrophobic antioxidant in cell membranes and circulating lipoproteins. Its antioxidant function is strongly supported by the regeneration promoted by vitamin C α-Tocopherol exhibits strong antioxidant capacity *in vitro* and has been shown to inhibit LDL oxidation (Wallert et al., 2014).

In addition, α-tocopherol shows a remarkable anti-inflammatory action by inhibiting, for example, cyclooxygenase-2 (COX2) (O’Leary et al., 2004). Next to its antinflammatory and antioxidative properties, vitamin E shows other properties, such as the modulation of the expression of genes encoding proteins involved in signaling (Cardenas and Ghosh, 2013). In addition vitamin E is also involved in the uptake, transport and degradation of tocopherols, as well as the uptake of lipoproteins and the storage and export of lipids such as cholesterol (Cardenas and Ghosh, 2013).

The beneficial effects of vitamin E dietary intake have been described (O’Leary et al., 2004; Hanson et al., 2015), whereas the data concerning vitamin E supplementation are controversial. An old randomized controlled trial by Stephens et al. (1996) showed that in patients with CAD, 1 year of α-tocopherol supplementation reduced the rate of non-fatal myocardial infarction. However, very few human studies have confirmed the efficacy of vitamin E supplementation in aging-associated diseases, and most of them focused on the role of vitamin E supplementation in influencing aspects of aging phenotypes, such as oxidative stress and inflammation biomarkers. In this specific context some investigations, performed both in animals models and in humans, effectively demonstrated benefits of vitamin E supplementation (Iuliano et al., 2000; Navarro et al., 2005; Abdala-Valencia et al., 2012), while others showed negative impact (Bjelakovic et al., 2012), or no effect at all (Morley and Trainor, 2001; Hemilä and Kaprio, 2011).

Two large randomized trials (Yusuf et al., 2000; Lonn et al., 2005) investigated the impact of vitamin E supplementation on CVDs and cardiovascular events in patients at high risk. The “Heart Outcomes Prevention Evaluation (HOPE)” analyzed the efficacy of a treatment with vitamin E in preventing cardiovascular outcomes in 9,541 patients with CVD or diabetes in addition to at least one other risk factor (Yusuf et al., 2000). This study, with mean follow-up of 4.5 years, showed that vitamin E did not reduce the incidence of cardiovascular events when compared to placebo, thus suggesting the lack of an evident beneficial effect exerted by this antioxidant (Yusuf et al., 2000).

The “HOPE-TOO” (Lonn et al., 2005) an extension of the HOPE study, was aimed at assessing whether longer duration of the treatment with vitamin E could prevent cancer and/or CVD during a follow-up of 7 years. The HOPE-TOO, involving 7,030 patients, confirmed that administration of 400 IU of vitamin E had no evident impact either on cancer outcomes or on major cardiovascular events and death. Furthermore, during the HOPE-TOO study, the investigators advanced the hypothesis that vitamin E supplementation might even be responsible to increase the risk of heart failure (Lonn et al., 2005).

Another clinical trial explored the effect of vitamin E on the development of chronic heart failure (CHF) in 8,415 post-infarction patients without CHF at baseline (Marchioli et al., 2006). The authors found that vitamin E treatment was associated with a 50% increase of CHF in patients with left ventricular dysfunction, thus confirming the conclusion raised by the HOPE trial investigators.

More recently, Devaraj et al. (2007) evaluated the effect of a high dose of α-tocopherol (1,200 IU/die for 2 years) in CAD patients with high levels of oxidative stress. The authors demonstrated that vitamin E supplementation lowered plasmatic levels of inflammation markers, such as high-sensitivity C-reactive protein and tumor necrosis factor-alpha, and the levels of oxidative stress biomarkers, such as plasmatic oxidized LDL, urinary F2-isoprostanes and monocytes superoxide anion concentrations (Devaraj et al., 2007). However, α-tocopherol supplementation failed to induce any change in intima-media thickness of carotid arteries and no significant differences in cardiovascular events were observed between patients treated with vitamin E and those with placebo (Devaraj et al., 2007).

As previously underlined, vitamins E and C have been frequently used in combination in clinical trials concerning aging-associated diseases. The “Women’s antioxidant Cardiovascular Study (WACS)” (Cook et al., 2007) investigated the effect of vitamins C, E and β-carotene (alone or in combination) in preventing cardiovascular events in 8,171 patients with either a history of CVD or at least three cardiovascular risk factors, and during an average 9.4 year follow-up (Cook et al., 2007). Results from WACS, as in the case of other antioxidant trials performed with women, failed to find any preventive effects of the antioxidants used on CVD.

The Physicians’ Health Study II (PHS II) (Sesso et al., 2008) randomized trial investigated instead the effects of vitamins E and C in the prevention of CVD in men during a mean follow-up of 8 years. This trial did not evidence any benefit from antioxidant supplementation on major CVD outcomes; moreover, vitamin E was associated with an increased risk of stroke (Sesso et al., 2008).

In a recent prospective study performed with 3,919 aged men, Wannamethee et al. (2013) showed that higher plasma levels of vitamin C, but not those of vitamin E, are inversely associated with cardiovascular risk factors, including blood lipids and blood pressure. Notably, whereas the dietary intake of vitamin C did not exert any influence, the dietary intake of vitamin E was significantly correlated with increased risk of HF (Wannamethee et al., 2013). The authors of this interesting investigation suggested that the reason for the association between vitamin E intake and HF might depend by the fact that vitamin E (α-tocopherol) may become a pro-oxidant in an environment characterized by high oxidative stress, such as an aged biological system (Wannamethee et al., 2013).

Resveratrol (3, 4′, 5-trihydroxystilbene) is a phytoalexin that belongs to the stilbene class of compounds, abundant in many plants, such as peanuts, blueberries, pine nuts and grapes where it mainly accumulates in a glycosylated form, and that is synthesized in response to fungal infection and to some environmental stresses like climate, ozone and ultraviolet irradiation (Harikumar and Aggarwal, 2008).

Resveratrol appears to modulate numerous cell-signaling pathways through the regulation of different molecular targets including the AMP-regulated kinase AMPK and the NAD-dependent deacetylase Sirt-1 (Yun et al., 2014; Conti et al., 2015a). The variety of molecular mechanisms mediated by this compound translates into a plethora of biological actions, primarily, antioxidant and anti-inflammatory effects. Resveratrol is a good antioxidant and blocks *in vitro* LDL oxidation, a biological phenomenon associated with the risk of coronary heart disease and myocardial infarction (Khurana et al., 2013). In rodents, resveratrol supplementation has been shown to decrease cardiovascular risk factor, including blood lipids and VCAM-1, to improve cardiovascular function and physical capacity and to decrease inflammation in the vasculature of aged animals leading to improved vascular function (Gliemann et al., 2013).

The anti-inflammatory properties of resveratrol have been proved by several *in vitro* experiments. For instance, resveratrol was showed to suppress NF-κB activity induced by beta-amyloid in PC12 neuron cell lines, (Jang and Surh, 2003) and to reduce the production of IL-1 beta and TNF-alpha induced by LPS or beta-amyloid in the microglia (Capiralla et al., 2012; Zhong et al., 2012), suggesting a neuroprotective effect that has also been confirmed in cellular models of neurodegenerative disorders, such as Parkinson’s and Alzheimer’s diseases (Albani et al., 2009). Resveratrol anti-inflammatory effect has been demonstrated also *in vivo*, i.e., in an animal model of asthma in which this molecule mitigated structural airway remodeling (Royce et al., 2011) or in rats with LPS-induce liver failure where resveratrol improved hepatotoxic markers by multiple mechanisms such as downregulation of NOS-2, and modification of oxidative stress parameters (Farghali et al., 2009).

Despite the promising results reported *in vitro* (Zhang et al., 2011; Montesano et al., 2013) and in animal models (Saleh et al., 2014), few studies have been performed directly in humans and the results obtained are not quite convergent.

Recent studies underlined the importance of patient selection in evaluating the potential therapeutic effects of resveratrol. Recently, Carrizzo et al. (2013) conducted an *ex vivo* study to investigate the effects of resveratrol on superior thyroid artery obtained from 59 patients (63 years of mean age) with hypertension and dyslipidemia, and found that resveratrol reduced ED via modulation of NO metabolism and attenuation of vascular oxidative stress. Interestingly, resveratrol failed to exert any effect in vessels from patients without hypertension or dyslipidemia (Carrizzo et al., 2013). A differential effect of resveratrol influenced by the initial health status was also suggested by a recent meta-analysis by Liu et al. (2014) which highlighted that resveratrol improves glucose control and insulin sensitivity in diabetic patients but does not affect glycemic values in non-diabetic subjects.

In a recent work published by Gliemann et al. (2013), the authors tested for the first time the combined effect of exercise training (ET) and resveratrol on vascular function in aged humans. In this trial 27 healthy physically inactive aged men were randomized into 8 weeks of daily intake of either 250 mg of trans-resveratrol or of placebo and were subjected to concomitant high-intensity ET (Gliemann et al., 2013).

The main aim of the study was to confirm if oral resveratrol supplementation improved the positive cardiovascular adaptations to ET in aged subjects by specifically increasing sirtuin 1 (SIRT1) mediated signaling and by promoting the endogenous antioxidant system. Interestingly, results showed that, whereas ET effectively improved several cardiovascular health parameters in aged men, concomitant resveratrol supplementation somehow blunted most of these effects leading, among others, to a significantly lower improvement in the training-induced increase in maximal oxygen uptake (Gliemann et al., 2013).

Curcumin is a lipophilic bioactive phenol derived from the rhizome of *Curcuma longa*, which shows low solubility and stability in aqueous solution. It is contained in culinary curry and used as a coloring agent in food (Bhullar et al., 2013). Orally ingested curcumin is metabolized into the active metabolite tetrahydrocurcumin by a reductase found in the intestinal epithelium (Sadowska-Bartosz and Bartosz, 2014). Extensive research during the last few decades has suggested a strong therapeutic and pharmacological potential of this molecule as antioxidant, antimutagenic, antiprotozoal and antibacterial agent (Bhullar et al., 2013).

Curcumin strong medicinal properties are also associated with reported anti-cancer and neuroprotective effect such as in Alzheimer disease (Brondino et al., 2014). A hormetic mechanism of action of this compound is suggested from studies showing that expression levels of the stress response protein Heme Oxygenase-1 (HO-1) were increased in cultured hippocampal neurons treated with curcumin (Scapagnini et al., 2006). Moreover, this phenolic compound has been shown to reverse chronic stress-induced impairment of hippocampal neurogenesis and increase expression of brain-derived neurotrophic factor (BDNF) in an animal model of depression (Xu et al., 2007). Several studies also showed that curcumin interacts with NF-κB, and through this interaction exerts protective function also in the regulation of T-cell-mediated immunity (Kou et al., 2013). Recently González-Reyes et al. (2013) identified curcumin as a neuroprotector against hemin, the oxidized form of heme, which induced damage in primary cultures of cerebellar granule neurons of rats. In this study, a pretreatment of the neurons with 5–30 μM curcumin increased by 2.3–4.9 fold HO-1 expression and by 5.6–14.3-fold Glutathione (GSH) levels. Moreover, 15 μM curcumin lowered by 55% the increase in ROS production, by 94% the reduction of GSH/glutathione disulfide ratio, and by 49% cell death induced by hemin. Furthermore, curcumin induced the translocation into the nucleus of nuclear factor related factor-2 (Nrf2), thereby stimulating an inflammatory and antioxidant response against hemin-induced neuronal death (González-Reyes et al., 2013).

Curcumin effects on both the arterial endothelial function and the central arterial compliance was recently evaluated in post-menopausal women that underwent a daily ingestion of 150 mg of curcumin (Akazawa et al., 2012). In 32 post-menopausal women the Flow Mediated Dilation (FMD) measured arterial endothelial function, before and after 8 weeks of curcumin ingestion or ET. After this time, the authors observed that FMD increased significantly both in the exercise and curcumin groups, whereas no significant change in FMD was detected in the control group (Akazawa et al., 2012). The results obtained suggested that a regular ingestion of curcumin could improve endothelial function and might be a potential alternative treatment for patients who are unable to exercise. In a different study performed by the same group (Akazawa et al., 2013) and involving this time 51 post-menopausal women, the effects of curcumin ingestion alone and in combination with aerobic ET on central arterial compliance were evaluated. In this case also, the regular ingestion of curcumin, as the ET alone, significantly increased carotid arterial compliance in the group analyzed. Interestingly, the combination of ET and curcumin ingestion, differently from what observed with resveratrol (Gliemann et al., 2013), led to a cumulative beneficial effect in the improvement of the arterial compliance (Akazawa et al., 2013).

Hydroxytyrosol is an ortho-diphenol (a catechol) abundant in olive, fruits and extra virgin olive oil (Waterman and Lockwood, 2007). This compound, due to its catecholic structure, shows a marked antioxidant activity and is able to scavenge oxygen and nitrogen free radicals, inhibit LDL oxidation, platelet aggregation and endothelial cell activation, and protects DNA from oxidative damage (Waterman and Lockwood, 2007; Notomista et al., 2011; Bulotta et al., 2014). Hydroxytyrosol is also a metal chelator and is able to scavenge the peroxyl radicals and break peroxidative chain reactions producing very stable resonance structures (Bulotta et al., 2014). Interestingly, scavenging activity of hydroxytyrosol has also been demonstrated with respect to hypochlorous acid (HOCl) (Visioli et al., 1998) a potent oxidant produced *in vivo* at the site of inflammation, a phenomenon which may be critical for the protection from atherosclerosis, since HOCl can oxidize the apoproteic component of LDL. Moreover, it has been recently reported (Giordano et al., 2014) that hydroxytyrosol is endowed with the ability to modulate an adaptive signaling pathway activated after endoplasmic reticulum (ER) stress and to improve ER homeostasis itself.

The antioxidant activity of hydroxytyrosol seems to be related *in vivo* to its high bioavailability: various studies have in fact documented a high degree of absorption of this compound, which is fundamental to exert its biological activities (Bulotta et al., 2014). Several studies, mostly performed in cell and animal models, have suggested beneficial effects of hydroxytyrosol in the prevention or treatment of chronic and degenerative diseases, especially CVD and cancer (Facchini et al., 2014). Most of the studies currently presented in literature on hydroxytyrosol are performed *in vitro* on cultured eukaryotic cells and very few are the clinical trials performed in humans and more specifically on elderly people. One of the main reasons is probably the fact that purified hydroxytyrosol is still very expensive, which hampers its use for long-lasting trials. Currently, the attention of the scientific community is focused more on the effect of olive oil supplementation on health, but olive oil is a complex mixture containing variable amounts of triacylglycerols, fatty acids and polyphenols (Waterman and Lockwood, 2007), thus no conclusive hypothesis of the use of purified hydroxytyrosol can yet be drawn from these studies.

Oliveras-López et al. (2013) evaluated the effects of daily consumption of extra virgin olive oil in 62 subjects aged 65–96 years. After a 6-week**s** daily intake of polyphenol-rich olive oil with high oleuropein derivative contents, the authors found a significant improvement in lipid profiles, including a reduction of total cholesterol and a significant increase of HDL levels. Moreover, in the same subjects, an increase of serum total antioxidant capacity, and a concomitant significant increase of catalase in erythrocytes and decrease in superoxide dismutase and glutathione peroxidase activities were also observed (Oliveras-López et al., 2013).

Coenzyme Q10 (CoQ10), referred to as ubiquinol in its most active (95%) and reduced form (Q10H2), is a lipophilic molecule present in the membranes of almost all human tissues, and essential for the respiratory transport chain (Onur et al., 2014). The side chain serves to keep the molecule anchored in the inner mitochondrial membrane, and the quinone ring, which is easily and reversibly reduced to the quinol form, enables it to fulfill its function of transferring electrons from complexes I and II to complex III in the respiratory chain, ultimately resulting in the reduction of oxygen to water and the generation of ATP (Nowicka and Kruk, 2010; Laredj et al., 2014). CoQ10 is also capable of recycling and regenerating other antioxidants such as α-tocopherol and ascorbate. CoQ10 has also been identified as a modulator of gene expression, inflammatory processes and apoptosis (Bhagavan and Chopra, 2007). The quinol prevents lipid peroxidation by inhibiting the initial formation and propagation of lipid peroxy radicals, and in the process it is oxidized to the quinone and H_2_O_2_ is produced. In addition, it has been shown to protect proteins from oxidation by a similar mechanism (Forsmark-Andrée et al., 1995), and to prevent oxidative DNA damage such as strand breakages. As well as its role in the cellular membranes, CoQ is also believed to function in the blood to protect lipoproteins such as very low density (VLDL), low density (LDL) and high density (HDL) lipoproteins from oxidation (Bentinger et al., 2007). Current evidence suggests that CoQ has a number of independent anti-inflammatory effects (Schmelzer et al., 2007). It has been shown to reduce the secretion of pro-inflammatory cytokines in monocytes and lymphocytes after an inflammatory stimulus by influencing the expression of NF-κB-dependent genes (Schmelzer et al., 2009; Bentinger et al., 2010). Furthermore, dietary supplementation with CoQ10 has been reported to improve ED in patients with diabetes by up-regulating nitric oxide production (patients received 200 mg CoQ10/day for 12 weeks) (Watts et al., 2002), and to decrease hepatic inflammatory stress caused by obesity in mice (Sohet et al., 2009).

Coenzyme Q10 supplementation at 300 mg/day was reported to significantly enhance antioxidant enzymes activities and lower inflammation in patients who have CAD during therapy with statins (Lee et al., 2013). Statins can effectively lower CoQ synthesis as they inhibit 3-hydroxy-3-methylglutaryl Coenzyme A reductase (HMG-CoA reductase), the rate-limiting enzyme in the pathway of cholesterol synthesis which includes the formation of the isoprenoid units required to produce CoQ (Goldstein and Brown, 1990). Moreover, CoQ levels may be pathologically modified in conditions associated with oxidative stress and in aging (Potgieter et al., 2013; Botham et al., 2015).

Data presented in literature on CoQ10 supplementation are heterogeneous and involve a very large number of pathologies. As for HF, no conclusions can be drawn on the benefits or harms of coenzyme Q10 as trials published in literature lack fundamental information concerning clinically relevant endpoints (Madmani et al., 2014). More in detail, reports on the effect of CoQ10 in diseases depending on oxidative stress in elderly people are scarce. In a recent study (Bloomer et al., 2012), 15 exercise-trained individuals (10 men and 5 women; 30–65 years) received 300 mg of reduced CoQ10 per day or a placebo for 4 weeks in a random order, double blind, crossover design. Treatment with CoQ10 resulted in a significant increase in total blood CoQ10 and reduced blood CoQ10, but did not translate into improved exercise performance or decreased oxidative stress (Bloomer et al., 2012).

## Hormesis and Genetic Variability Influence on the Outcomes of Antioxidants Supplementation

As previously reported, clinical trials involving the use of antioxidants supplementation often show conflicting results and lead to dangerous misconceptions, either too positive or too negative, on the use of these molecules in the treatment of several aging-associated diseases. Amid this debate, the first aspect that should be considered is that there are several limitations concerning FRTA, the basic hypothesis on which the antioxidants supplementation therapies are mainly based. This theory, as already underlined, suggests a linear dose-response relationship between increasing amounts of ROS and biological damages, which potentially culminates in diseases and mortality. Therefore, oxidative stress should represent the main driving force of aging and a major determinant of lifespan (Sadowska-Bartosz and Bartosz, 2014). To date, many investigations have urged to reexamine FRTA leading to a modernized view of this theory that takes also in account the so-called “mitohormesis.” According to this concept, a large amount of ROS causes detrimental effects on the cells, whereas low or moderate levels of ROS may exert an opposite effect improving biological outcomes (Ristow and Schmeisser, 2014; Yun and Finkel, 2014). The beneficial effects of caloric restriction (CR) and ET are a good example because they can be considered both as oxidative stressors or inducer of the endogenous antioxidant system activation by favoring a transient cellular increase of ROS (Corbi et al., 2012). Many independent investigations raised the possibility that an initial induction of ROS triggered by CR promotes an adaptive stimulation of antioxidant enzymes at the steady state, consequentially CR is now considered as the first example of mitohormesis (Agarwal et al., 2005; Schulz et al., 2007; Mesquita et al., 2010; Zarse et al., 2012). CR likely induces an adaptive hormetic response through different molecular pathways, one of these involving sirtuins, a family of NAD^+^-dependent deacetylases conserved from yeasts to humans (Banerjee et al., 2012). SIRT1, the first member of sirtuins characterized in humans, plays a crucial role in inducing mitochondrial biogenesis and mediating oxidative stress response through a number of proteins that promote the expression of antioxidant genes, such as peroxisome proliferator-activated receptor (PPAR) gamma coactivator-1 alpha 5 (PGC-1α) (St-Pierre et al., 2006) and Forkhead transcription factors member, FOXO3a. SIRT1 interacts with FOXO3a in cells in response to oxidative stress increasing FOXO3 ability to induce cell cycle arrest and resistance to oxidative stress and, at the same time inhibiting FOXO3 ability to induce cell death (Brunet et al., 2004). Ferrara et al. (2008) demonstrated that exercise-induced increase in SIRT1 activity in the heart of aged rats caused an increase in the expression of FOXO3a and an up-regulation of FOXO3a targets involved in the oxidative stress response, including SOD and catalase.

Exercise training, as CR, is to date considered an intervention triggering a cellular hormetic adaptation (Radak et al., 2005; Ji et al., 2006). Physical inactivity is indeed one of the major risk factors for CVD, neurodegenerative disorders and many other diseases; consequentially, regular physical exercise exerts health promoting effect on such clinical conditions and in general on aging-related diseases (Hu et al., 2001; Conti et al., 2012b; Brown et al., 2013). Exercise is strictly correlated to enhanced mitochondrial biogenesis and increased production of ROS and may promote longevity through pathways common to those of CR (Lanza et al., 2008). However, the benefits linked to ET strictly depend on the type and workload of exercise and, in particular, overtraining can result in maladaptation and possibly cellular damage (Alessio and Goldfarb, 1988; Chevion et al., 2003; Conti et al., 2012a, 2013). ET has been reported to activate PGC-1α, which controls mitochondrial gene expression by a variety of transcription factors (Nikolaidis and Jamurtas, 2009). This regulation culminates in enhanced oxygen consumption in muscle fibers, which, in turn, promotes ROS generation. Moreover, beyond skeletal muscle, other tissues, such as blood, heart and lung, represent a source of ROS during exercise (Nikolaidis and Jamurtas, 2009). Concomitantly to enhanced ROS production, regular exercise leads to the up-regulation of several antioxidant enzymes, including SODs, catalase and glutathione peroxidase, reinforcing the concept that a certain amount of ROS is necessary for exercise health-promoting effects (Nikolaidis and Jamurtas, 2009).

It is not surprising, then, that both older and recent studies showed that purified antioxidants supplementation might be inadequate or even damaging for athletes, as they seem to abolish ET benefits, including prevention of certain diseases. A very interesting study by Ristow et al. (2009) investigated whether exercise could promote insulin-sensitizing abilities in a ROS-dependent manner in healthy humans. The authors measured insulin sensitivity by glucose infusion rate (GIR) and the amount of ROS within skeletal muscle of trained subjects (previously untrained) in the presence or absence of antioxidant supplementation with vitamin E and vitamin C. As expected, ET induced ROS formation, which was counteracted by the antioxidant treatment. However, concomitantly to the increase of TBARs, ET was able to stimulate the expression of antioxidant molecules, such as SOD and GPx and induced an increase of GIR; these effects were also inhibited by antioxidants supplementation. The conclusion of this study was that a transient increase of oxidative stress may contribute to prevent insulin resistance and type 2 diabetes and, most importantly, antioxidant supplementation may abrogate these results (Ristow et al., 2009).

In addition to hormesis another aspect that should be considered for the conflicting results obtained in the clinical trials is the genetic background of the patients enrolled in the studies. In the last decade an increasing number of studies have suggested that longevity depends not only on life style habits but also on the genetic background. Oxidative stress response is one of the most evolutionary conserved pathways involved in determination of lifespan from yeast to humans (Vijg and Suh, 2005; vB Hjelmborg et al., 2006) and, indeed, genome wide association studies (GWAS) have identified genetic determinants associated to the levels of circulating antioxidants, which could be linked to human diseases (Ahn et al., 2010). A GWAS authored by Major et al. (2012) revealed that three single nucleotide polymorphisms (SNPs), two located in genes involved in vitamin E transport and metabolism (BUD13 and CYP4F2), and one in NKAIN3, the gene encoding a Na+/K+ transport membrane protein, are associated with response to vitamin E supplementation in humans. The authors concluded that genetic variation contributes to the variability of serologic vitamin E status and may have potential application in determining the regimen of antioxidant supplementation required in complex diseases such as CVD and diabetes (Major et al., 2012). Very interesting data concern Haptoglobin (Hp), a protein encoded by a polymorphic gene with 2 common alleles denoted 1 and 2, which counteracts the increase of ROS induced by hemoglobin activity (Sadrzadeh et al., 1984). As previously reported, the HOPE trial, which investigated the potential protective effect of vitamin E in cardiovascular patients, showed that treatment with vitamin E had no effect on cardiovascular outcomes in patients at high risk for cardiovascular events (Yusuf et al., 2000). Later, Milman et al. (2008) verified such results moving from the hypothesis that HOPE study failed to prove the benefit of vitamin E supplementation because of the inadequate selection of patient genotype. To this end, the authors planned a prospective double-blinded clinical trial in a subgroup of individuals from the HOPE study with type 2 diabetes and found that vitamin E supplementation was effectively able to reduce cardiovascular events in patients with the Hp 2-2 genotype (Milman et al., 2008). Other studies confirmed the impact of Hp genotyping in determining potential benefits from antioxidant therapy, and strongly supported the efficacy of a pharmacogenomic strategy to personalize and fine-tune the treatment with vitamin E in patients with type 2 diabetes (Blum et al., 2010).

## Conclusion

Redox state homeostasis in living systems is very complex and life style factors undeniably concur in determining the impact of changes in oxidative stress response in both unhealthy and healthy subjects.

A large part of studies investigating the effectiveness of antioxidant supplementation therapy in humans raised contrasting results. This is due to many aspects among which the often-limited statistic power of the studies, the patient genetic background, the bioavailability of the molecules used, and the non-specific effects that antioxidants might have in the human body, should be taken into account.

Mainly in the elderly, the clinical trials conducted so far often suffer from an incorrect initial selection of the patients. Further investigations should be planned to improve patients selection by performing, for example, quantitative characterizations of the redox state for each individual and taking into account both the individual patient demand and genetic background.

In addition it is worth to underline that, when dealing with either natural or synthetic antioxidants, clinical trials should consider other two important aspects. First, antioxidants bearing different functional moieties can be profoundly diverse in terms of chemical structure and mode of action; therefore, it should be recommended to identify the right antioxidant to treat a specific pathological condition (Bast and Haenen, 2013). Secondly, the validity of the biomarkers used to determine the effects of antioxidants on human health are still under debate (van Ommen et al., 2009). Antioxidants, in fact, might be responsible of subtle effects specific for human health optimization and/or disease prevention, which are processes that can be very different in many aspects from disease onset and progression.

## Author Contributions

VC, VI, and GC conceived and designed the review and wrote the paper; GR and VM performed the bibliographic research; FD and AD edited the manuscript; AF contributed to write the paper.

## Conflict of Interest Statement

The authors declare that the research was conducted in the absence of any commercial or financial relationships that could be construed as a potential conflict of interest.
